# Integrated genomic and transcriptomic analysis reveals the activation of PI3K signaling pathway in HPV-independent cervical cancers

**DOI:** 10.1038/s41416-023-02555-w

**Published:** 2024-01-22

**Authors:** Yi Wang, Misi He, Tiancong He, Xueyan Ouyang, Xuxia Shen, Wanling Shi, Shengling Huang, Libing Xiang, Dongling Zou, Wei Jiang, Huijuan Yang

**Affiliations:** 1 Department of Gynecological Oncology, Fudan University Shanghai Cancer Center, Fudan University, Shanghai, 200032 China; 2 Department of Oncology, Shanghai Medical College, Fudan University, Shanghai, 200032 China; 3 Department of Gynecologic Oncology, Chongqing University Cancer Hospital & Chongqing Cancer Institute & Chongqing Cancer Hospital, Chongqing, 400030 China; 4 Chongqing Specialized Medical Research Center of Ovarian Cancer, Chongqing, 400030 China; 5 Organoid Transformational Research Center, Chongqing Key Laboratory of Translational Research for Cancer Metastasis and Individualized Treatment, Chongqing University Cancer Hospital, Chongqing, 400030 China; 6 Department of Surgical Oncology, Minhang Branch, Fudan University Shanghai Cancer Center, Shanghai, 200240 China; 7 Department of Pathology, Fudan University Shanghai Cancer Center, Fudan University, Shanghai, 200032 China; 8 Cancer Institute, Fudan University Shanghai Cancer Center, Shanghai, 200032 China; 9 Department of Gynecologic Oncology, Zhongshan Hospital, Fudan University, Shanghai, 200032 China

**Keywords:** Cervical cancer, RNA sequencing

## Abstract

**Background:**

HPV-independent cervical cancers (HPV-ind CCs) are uncommon with worse prognosis and poorly understood. This study investigated the molecular characteristics of HPV-ind CCs, aiming to explore new strategies for HPV-ind CCs.

**Methods:**

HPV status of 1010 cervical cancer patients were detected by RT-PCR, PCR and RNA-sequencing (RNA-seq). Whole exome sequencing (WES) and RNA-seq were performed in identified HPV-ind CCs. The efficacy of PI3Kα inhibitor BYL719 in HPV-ind CCs was evaluated in cell lines, patient-derived organoids (PDOs) and patient-derived xenografts (PDXs).

**Results:**

Twenty-five CCs were identified as HPV-ind, which were more common seen in older, adenocarcinoma patients and exhibited poorer prognosis as well as higher tumor mutation burden compared to HPV-associated CCs. HPV-ind CCs were featured with highly activated PI3K/AKT signaling pathway, particularly, PIK3CA being the most predominant genomic alteration (36%). BYL719 demonstrated superior tumor suppression in vitro and in vivo. Furthermore, HPV-ind CCs were classified into two subtypes according to distinct prognosis by gene expression profiles, the metabolism subtype and immune subtype.

**Conclusions:**

This study reveals the prevalence, clinicopathology, and molecular features of HPV-ind CCs and emphasizes the importance of PIK3CA mutations and PI3K pathway activation in tumorigenesis, which suggests the potential significance of PI3Kα inhibitors in HPV-ind CC patients.

## Introduction

Cervical cancers (CCs) are one of the most threatening female reproductive system malignancies worldwide and rank as the fourth most common and cause of cancer death in women, accounting for ~569,000 new cases and 311,000 deaths every year [1, 2]. Human papillomavirus (HPVs) has long been identified as the key causative factor for the development of CCs [3, 4]. Once high-risk HPVs have infected cervical basal cells, they express the viral E6 and E7 gene products, which bind to tumor suppressor proteins p53 and pRB, disrupting normal cell cycle regulation, accelerating cell proliferation, and increasing the likelihood of malignant transformation [5]. Current estimates of HPV prevalence in CCs patients vary from 85% to 99% in different cohorts [3, 6–8]; however, with the widespread adoption of CCs screening and endorsement of HPV vaccination [9], the incidence of HPV-associated CCs (HPV-asso CCs) will predictably decrease. Consequently, it is of importance to understand the mechanism of the occurrence and development of HPV-independent cervical cancers (HPV-ind CCs), which will be helpful to the early diagnosis and treatment.

It is suggested that HPV-ind CCs present a biologically distinct subgroup with different molecular characteristics and poorer prognosis compared to HPV-asso CCs [8, 10, 11]. Nicolás showed a higher proportion of abnormal staining patterns of p53 expression and p16 overexpression in HPV-ind CC [12]. A study from The Cancer Genome Atlas (TCGA) enrolled 9 HPV-ind CCs revealed a higher rate of genomic mutations in KRAS, ARID1A and PTEN [10], suggesting potential driver events in HPV-ind CCs. Other studies have also emphasized the role of long noncoding RNAs (lncRNAs) on promoting tumor growth and recurrence [13, 14]. However, the existing studies are limited to small-scale, substandard HPV screening modalities and, most importantly, there is a lack of comprehensive studies to guide treatment.

Thus, in the current study, we aimed to characterize the molecular landscapes of HPV-ind CCs using whole exome sequencing (WES) and RNA-sequencing (RNA-Seq), through which we identified the activation of *Pi3k* pathway as the possible pathogenic mechanism, indicating PI3K inhibitors as a therapeutic strategy for HPV-ind CCs.

## Methods

### Study design and patient eligibility

The study comprised two sets of CCs patients: patients from Shanghai Cancer Center (SHCC) and The Cancer Genome Atlas (TCGA) cervical squamous cell carcinoma and endocervical adenocarcinoma (CESC) cohort. For the cohort from SHCC, ethical approval was granted by the Ethics Committee of Fudan University Shanghai Cancer Center (NO.050432-4-1212B), and informed consent was obtained from each participant. One thousand ten samples from our previous cervical cancer banks (*n* = 1015) were enrolled in the current study, because 5 of these samples had insufficient DNA. The inclusion criteria and clinical data retrieval were performed as described in our previous publications [15–17]. The molecular and clinical data of the TCGA-CESC cohort were downloaded from the data portal of Genomic Data Commons (GDC, https://portal.gdc.cancer.gov/).

### The detection of HPV infection

**Fig. 1:**
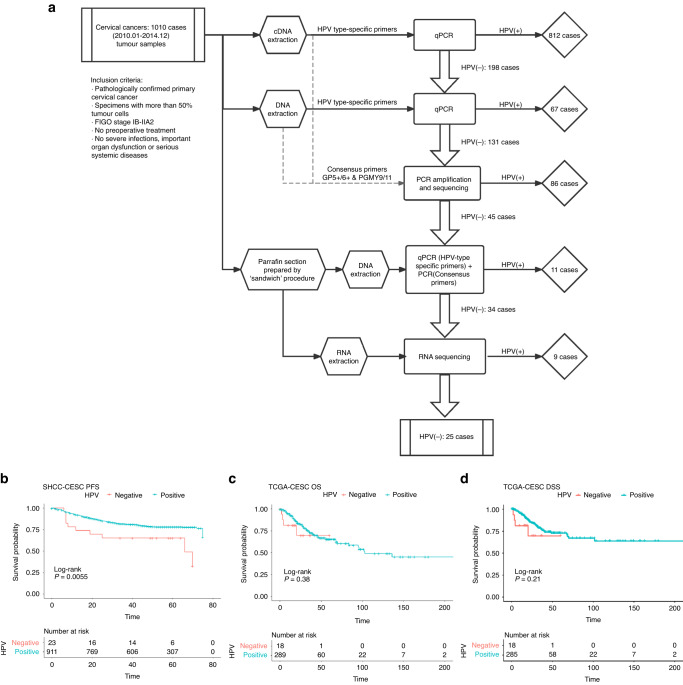
The screening procedure and survival plot of CCs. **A** Schematic diagram of the screening procedure and the results for HPV-ind CCs. **B** Kaplan-Meier plots for PFS of data from SHCC. Kaplan-Meier plots for OS (**C**) and DSS (**D**) of data from TCGA.

The HPV screening process was performed by real-time PCRs for 7 high-risk and other common HPV subtypes, and then validated by RNA-Seq. The flow-process diagram and results are shown in Fig. 1A. More detailed methods are provided in Supplemetary materials.

### Whole-exome sequencing (WES)

DNA of tumor tissues and paired normal tissues from 21 HPV-ind CCs patients was subjected to WES. It was performed according to our previous study [18].

### PIK3CA mutation analysis by Sanger sequencing

Validation analyses of *PIK3CA* mutation status were performed using cDNA-based Sanger sequencing as previously described [15–17]. The primers and procedures are presented in Table S2.

### RNA-Seq analysis

All 53 CC samples and 4 PDXs samples were subjected to RNA sequence, which was performed as our previous study [19].

### Comparison of molecular characteristics between groups

Differentially expressed genes between HPV-asso CCs and HPV-ind CCs were identified by R package edgeR with a cutoff of false discovery rate (FDR) < 0.05 and fold change (FC) > 1. Then logFC was used in pre-rank GSEA analysis against Reactome, Kegg and hallmark gene sets by R package clusterProfiler to access the molecular features between two groups. A pathway was considered enriched at FDR < 0.25 and normalized enrichment score (NES) > 1.

### Identification of subtypes in HPV-ind CCs

Unsupervised hierarchical clustering of RNA-Seq data was performed using features selected based on the most variant median absolute deviation (MAD) and Cox regression model by the CancerSubtypes package, dividing the 25 HPV-ind CCs into distinct subgroups. The optimal number (*K* = 2) of clusters was chosen by the ConsensusClusterPlus package. The same features selected were used in clustering the TCGA validation set.

### Cell culture and drug treatment

Human squamous carcinoma of the cervix cell lines SiHa, MS751 and C-33A were obtained from American Type Culture Collection (ATCC). SiHa and MS751 cells were positive for HPV-16 and HPV-18, respectively, and C-33A cells were HPV-independent. All cell lines tested free of mycoplasma contamination were cultured in Dulbecco’s modified Eagle medium (DMEM) supplemented with 10% fetal bovine serum (FBS) (Gibco), 1% penicillin, and 1% streptomycin, and incubated at 37 °C with 5% CO_2_. The PI3Kα inhibitor BYL719 was purchased from Selleck (S2814)

### IC_50_ assay

Cells were seeded onto 96-well plates at a density of 6 × 10^3^ cells/well. A gradient concentration of BYL719 from 0.5 μM to 128 μM and a control of DMSO were added into wells after attachment. 10 μL of CCK-8 diluted in 90uL of DMEM medium was added to each well 72 h later and incubated for 2 h, followed by measurement of optical density at 450 nm (OD450). GraphPad was used to fit the data, generate dose-response curves and calculate IC_50_ values.

### Colony formation assay

Cells were seeded onto six-well plates at a density of 1 × 10^3^ cells/well and cultured for 24 h before drug treatment. Fresh medium containing 25 μM BYL719 was replaced every 3 days. At the end point, cells were washed with phosphate-buffered saline (PBS) and fixed with formalin followed by staining with 5% crystal violet. After capturing the images, colonies with over 50 cells were quantified by image J.

### Apoptosis and cell cycle assay

Cells were digested from plates after treatment with 25 μM BYL719 for 72 h, and then stained with Annexin-V and 7-AAD or PI for apoptosis or cell cycle distribution, respectively, following the manufacturer’s instructions (Cell Cycle Analysis Kit, Beyotime, Shanghai and Annexin V-PE/7-AAD apoptosis kit, MULTISCIENCES, Hangzhou). Cell cycle distribution and apoptosis were analyzed by flow cytometry.

### Western blot assay

Western blot was performed as previously described to evaluate the protein expression in cells between drug treatment groups [20]. The antibodies were diluted as following: BAX (1:1000, Cat# ab32503), BCL-2 (1:1000, Cat# ab32124), CDK2 (1:4000, Cat# ab32147), CDK4 (1:4000, Cat# ab108357), CDK6 (1:4000, Cat# ab124821), RAD51 (1:1000, Cat# ab133534), Cleaved caspase3 (1:2000, Cat# ab32042), γH2AX (1:2000, Cat# 9718), AKT (1:2000, Cat# 4685), pAKT Ser473 (1:1000, Cat# 4060), pAKT Thr308 (1:1000, Cat# 13038), pS6 S240/244 (1:2000, Cat# 5364), pS6 S235/236 (1:2000, Cat# 4858) and β-actin (1:2000, Cat# 4970).

### Patient-derived organoids (PDOs)

Cervical tumor tissues from consenting patients were first mechanically shredded with scalpels and then digested in collagenase + TryplE solution for 1–1.5 h in a 37 °C shaker. The cell suspensions were then washed three times with AdDF + ++ (Advanced DMEM/F12 supplemented with 1x Glutamax, 10 mM HEPES and penicillin–streptomycin), and erythrocytes were lysed with erythrocyte lysis buffer. Cells were filtered through a 100 μm nylon cell strainer and collected via centrifugation. Cells were subsequently embedded into basement membrane extracts and plated as 50 μl volume droplets on pre-warmed six-well suspension culture plates and allowed to solidify at 37 °C for 30 min prior to addition of medium.

### Patient-derived xenografts (PDXs)

The establishment and administration were detailed described in our previously study [21]. Four mice for HPV-asso PDXs and HPV-ind PDXs each were randomly divided into two groups when the volume of the tumor reached 100 to 300 mm^3^ and treated with BYL719 45 mg/kg (p.o.) and saline for 4 weeks, respectively. Ki-67, cleaved caspase3 and γH2AX antibodies were used to measure the corresponding protein expression by IHC staining.

### Quantification and statistical analysis

All analyses were performed in R software version 4.1.1 (http://www.r-project.org). The survival of HPV-asso and HPV-ind CCs patients were presented in Kaplan-Meier curve and compared by the Log-rank test using R package survminer and survival. Continuous data of clinical features and molecular features between groups were analyzed by Wilcoxon rank-sum test and categorical variables were analyzed by chi-square test (or Fisher’s exact test as indicated). Quantitative results from cell experiments were analyzed with Student’s *t* test in GraphPad. Two-tailed *P* values < 0.05 were considered statistically significant. Data were visualized using the R package ggplot2, with R package ggpubr for statistical analysis. Heatmaps were generated with the R package pheatmap.

## Results

### The clinicopathological association and prognosis analysis of HPV-ind CCs

HPV-ind CCs accounted for 2.48% (25/1010) in our cohort (Fig. 1A). Of all HPV-ind CCs, 11/25 (44%) were squamous cell carcinoma (SCC), 12/25 (48%) were classic adenocarcinoma (ADC), 1/25 (4%) was neuroendocrine tumor (NET) and 1/25 (4%) was peripheral primitive neuroectodermal tumor (PNET). The mean age of patients was 52.8 years old (52.8 ± 14.3). Patient characteristics are detailed in Table 1. HPV-ind CCs patients were older (52.8 vs. 47.6-year-old, *P* = 0.009) and more likely to be in the post-menopausal stage (60% vs. 36%, *P* = 0.027). They were more accompanied with parametrial involvement (16% vs. 5%, *P* = 0.049). ADC was more commonly observed in HPV-ind CCs than HPV-asso CCs patients (48% vs. 15%, *P* < 0.001). The TCGA dataset included 307 samples, of which 18 were HPV-ind and 289 were HPV-asso (Table 1). The clinical characteristics were basically consistent with those of our cohort except for menopause status, and the proportion of deaths.

**Table 1:** Patients’ characteristics bewteen HPV-independent and HPV-associated cervical cancers.

	*N* (%)	SHCC		*P* value^a^	*N* (%)	TCGA		*P* value^a^
		HPV-ind	HPV-asso			HPV-ind	HPV-asso	
		(*n* = 25)	(*n* = 972)			(*n* = 18)	(*n* = 289)	
Age, Mean (SD)				**0.009**				**0.014**
Mean	47.7	52.8	47.6		48.5	55.3	47.8	
SD	9.62	14.3	9.5		13.9	11.2	13.8	
Menopause status				**0.027**				0.894
Pre-menopause	629 (63%)	10	619		125 (60%)	10	115	
Post-menopause	368 (37%)	15	353		84 (40%)	8	76	
Histological subtypes				**<0.001**^**c**^				**0.001**
SCC	733 (74%)	11	722		254 (83%)	9	245	
ADC	154 (15%)	12	142		48 (15%)	6	42	
ASC	79 (8%)	0	79		5 (2%)	3	2	
Others	31 (3%)	2	29		0 (0%)	0	0	
FIGO stage				0.892				0.266
Stage I	472 (47%)	11	461		163 (54%)	7	156	
Stage II+	525 (53%)	14	511		137 (46%)	11	126	
Lymph node involvement				0.641				0.179^e^
Yes	297 (30%)	9	288		53 (29%)	1	52	
No	700 (70%)	16	684		127 (71%)	10	117	
Tumor Size				1				0.0502^e^
>4 cm	316 (32%)	8	308		159 (54%)	14	145	
≤4 cm	681 (68%)	17	664		133 (46%)	4	129	
LVSI^**d**^				0.685				1.000^e^
Yes	378 (38%)	11	367		80 (53%)	5	75	
No	614 (62%)	14	600		72 (47%)	5	67	
Depth of myometrial invasion			0.387^e^					
Whole-thickness	406 (41%)	13	393					
>1/2	325 (32%)	8	317					
≤1/2	266 (27%)	4	262					
Parametrial involvement				**0.049**^**e**^				0.123
Yes	57 (6%)	4	53		108 (36%)	10	98	
No	940 (94%)	21	919		193 (64%)	8	185	
PI3KCA mutation status				**0.006**				**0.002**^**e**^
Wild type	853 (86%)	16	837		205 (71%)	5	200	
Mutant	144 (14%)	9	135		84 (29%)	10	74	
Outcomes	199 (20%)	12	187	**0.001**^**e**^	72 (23%)	4	68	0.306^e^
Relapse	183 (19%)	12	171	**<0.001**^**a**^				
Death	117 (12%)	8	109	**0.005**^**a**^	72 (23%)	4	68	0.306^e^

^a^Wilcox test for age; *X*^2^ test for other characteristics.

^b^*SCC* Squamouscarcinoma, *ADC* Adenocarcinoma, *NEC* Neuroendocrine carcinoma, *ASC* Adenosquamous carcinoma.

^c^SCC vs. ADC.

^d^LVSI Lymphovascular invasion.

^e^Fisher exact test.

In the median follow up of 54 months (range: 1–75 months), HPV status was significantly associated with relapse (HPV-ind: 48% vs. HPV-asso: 18%, *P* < 0.001). Univariate analysis revealed a striking association between HPV status and patient survival, concordant with previous studies [7, 8, 22], HPV-ind CCs showed a worse outcome than HPV-asso CCs (5-year PFS: 52% vs. 82%, *P* = 0.001, 5-year OS: 68% vs. 88%, *P* < 0.001) (Fig. 1B; Table 2). Our multivariate analyses revealed that HPV-ind in cervical cancer was an independent predictor for poorer PFS (HR = 0.53, 95% CI: 0.28–0.99, *P* = 0.045) (Table 2). Similarly, poorer OS and PFS were observed in patients with HPV-ind CCs from the TCGA cohort, although statistical significance was not achieved. (Fig. 1C, D). Collectively, these results revealed that HPV-ind CCs formed a rare but more deadly type of CCs distinct from HPV-asso CCs.

**Table 2:** HPV status in cervical cancer predicted independently better PFS in univariate and multivariate analyses.

	Univariate analyses			Multivariate analyses		
Clinicopathologic characteristics	HR	95% CI	*P* value	HR	95% CI	*P* value
Age (>48 years)	1.19	0.900–1.574	0.223			
Postmenopausal	1.243	0.937–1.650	1.243			
Tumor sizes (>4 cm)	1.432	1.072–1.912	0.015	1.09	0.81–1.47	0.563
Depth of myometrial invasion (>1/2)	3.632	2.309–5.713	<0.001	2.12	1.40–3.46	**0.003**
Parametrial involvement	3.31	2.217–4.914	<0.001	1.47	0.96–2.27	0.078
LVSI	2.356	1.780–3.117	<0.001	1.62	1.18–2.21	**0.003**
Node status	3.405	2.572–4.508	<0.001	2.09	1.51–2.89	**<0.001**
Histological subtypes^a^	1.825	1.364–2.441	<0.001	2.13	1.02–1.86	**<0.001**
FIGO status	1.82	1.360–2.435	<0.001	1.38	1.02–1.86	**0.039**
HPV status^b^	0.407	0.222–0.748	0.004	0.53	0.28–0.99	**0.045**

*SCC* squamous cell carcinoma, *LVSI* lymphovascular invasion.

^a^Non-SCC vs. SCC.

^b^HPV positive vs. HPV negative.

### The PI3K pathway was highly activated in HPV-ind CCs

#### Genomic alterations

**Fig. 2:**
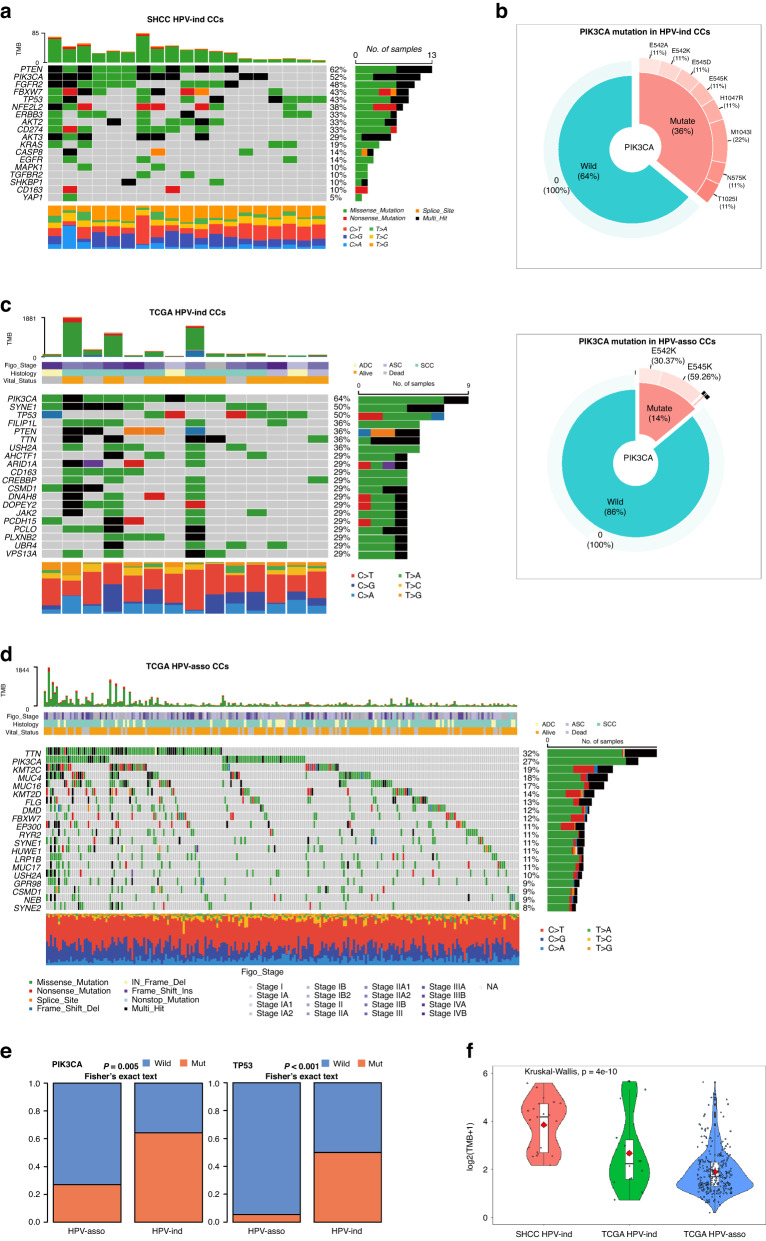
Mutation alterations of CCs. **A** Somatic mutations in HPV-ind CCs from the SHCC cohort. The upper panel shows the number of mutational events for each sample, and the bottom panel shows the transition/transversions type. **B** Sanger sequencing of *PIK3CA* mutation in SHCC cohorts. Somatic mutations in HPV-ind CCs (**C**) and HPV-asso CCs (**D**) from the TCGA cohort. The middle panel shows the clinical features of each sample. **E** Stacked bar chart of *PI3KCA* (left) and *TP53* (right) mutations in HPV-asso and HPV-ind CCs from the TCGA cohort. **F** Comparison of TMB between HPV-ind and HPV-asso CCs from both SHCC and TCGA cohort.

WES was performed to evaluate the genomic variations in 21 HPV-ind CCs (Fig. 2A). It showed that more PI3K pathway-related mutation were found: *PTEN* (62%), *PIK3CA* (52%), and *AKT2* (33%). We then analyzed the *PIK3CA* status of 25 HPV-ind CCs and 972 HPV-asso CCs, which had the detailed mutation information in exon9, and exon20 by Sanger sequencing in our previous study. Compared to HPV-asso CCs, HPV-ind CCs presented higher *PIK3CA* mutations (36% vs. 13%, *P* = 0.006, Fig. 2B). Other high-frequency mutations appeared in *FGFR2* (48%), *FBXW7* (43%) and *TP53* (43%) in HPV-ind CCs. Similarly, single nucleotide variation (SNV) from the TCGA dataset was analyzed for validation (Fig. 2C, D). It demonstrated that *TTN* (32%), *PIK3CA* (27%), *KMT2C* (19%), *MUC4* (18%), *MUC16*(17%) were the top 5 most frequent mutated genes in 274 HPV-asso CCs, while among 14 patients with HPV- CESC, *PIK3CA* (64%), *SYNE*1 (50%), *TP53* (50%), *PTEN* (36%), *TTN* (36%) sited the 5 most frequent mutations, depicting a different mutation signature. *PIK3CA, EP300, FBXW7, ARID1A, PTEN* were identified as significantly mutated genes (SMGs) in HPV-asso CCs, which is consistent with previous findings of CCs overall [10], however, they were *NDUFS1, PIK3CA, PTEN* and *TP53* in HPV-ind CCs. (Table S3, S4). Concordant with our cohort, HPV-ind CCs in the TCGA database had a higher mutation rate of *PIK3CA* and *TP53* (Fig. 2E, *PIK3CA* 9/14 64% vs. 74/274 27%, *p* = 0.005; *TP53* 7/14 50% vs. 15/274 5.5%, *P* < 0.001 Fisher’s Exact Test). Most mutations of *PIK3CA* located in the activating helical domain E542K and E545K (Fig. S1A), whereas mutations in *TP53* were scattered with no hotspots as reported in both HPV-asso and HPV-ind CESCs (Fig. S1B). No survival differences were observed between *PIK3CA* mutant and wild-type samples in either HPV-ind CCs or HPV-asso CCs, so as between *TP53* mutation status (Fig. S1C–H).

In addition, HPV-ind CCs demonstrated a higher tumor mutation burden (TMB) compared to HPV-asso CCs from the analysis of TCGA cohort (11.11 vs 3.84, *p* < 0.05, Fig. 2F). Due to lack of genomic information from WES in HPV-asso CCs patients, we analyzed the TMB only in SHCC HPV-ind CCs. It revealed that the TMB of HPV-ind CCs in our cohort was 16.20, which was significantly higher than that of HPV-asso CCs from the TCGA cohort.

Copy number variants (CNVs) were processed and analyzed by GISTIC2.0 in TCGA dataset. An average of 288.5 and 302.2 copy number variants were found for HPV-asso and HPV-ind CESCs, respectively (*P* = 0.91, Wilcoxon test), but a significant variance of 26.2 and 2.5 CNVs each for HPV-asso and HPV-ind CESCs were achieved after including CNVs restricted to *q* < 0.1 (Fig. S2A, B, *P* < 0.001, Wilcoxon test). It revealed 46 amplifications and 36 deletions in HPV-asso CESCs while only 1 amplification and 2 deletions in HPV-ind ones (Fig. S2C, D). The CNVs in HPV-ind CESC included amplification of 3q26.31 (*GHSR, FNDC3B*, 44.4%) and deletion of 5q11.2 (*PDE4D, PART1*, 27.8%) and 22q13.32 (*MAPK11, TYMP,* 27.8%), which displayed different variations with HPV-asso CCs. It is noteworthy that though 3q26.31 appeared in both HPV status, the distribution of its amplification seemed to be complementary to those without PIK3CA mutation in HPV-ind CESCs (Fig. S2E). As GHSR rarely expressed in CESCs, *FNDC3B* might play an important role in *PIK3CA* wild type HPV-ind CESCs.

#### Transcriptome analysis

**Fig. 3:**
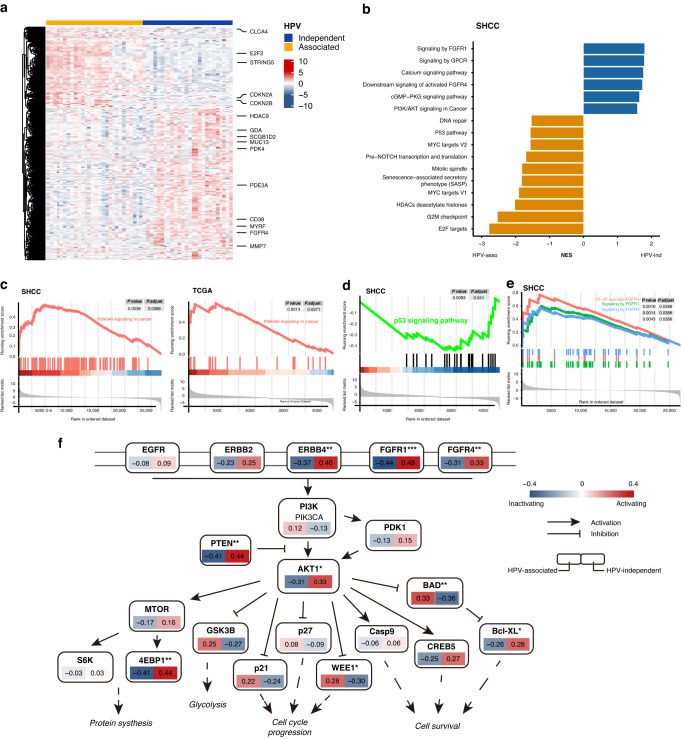
Transcriptome analysis revealed high activation of the PI3K/AKT pathway in HPV-ind CCs. **A** Heatmap of significantly differential expression in cervical cancers with several of the most variable mRNAs annotated on the right. **B** Bar plot shows cancer-related pathways enriched by GSEA between HPV-ind and HPV-asso CCs in SHCC cohort. GSEA plot displays that PI3K/AKT signaling was enriched in HPV-ind CCs in both cohorts (**C**) while TP53 signaling pathway was enriched in HPV-asso CCs (**D**). FGFR pathways were also enriched in HPV-ind CCs (**E**). **F** Relative levels of transcript of main effectors in the PI3K/AKT signaling pathway.

We further explored the correlation between *PIK3CA* mutation mediated PI3K pathway activation and HPV status in cervical cancers. RNA-Seq was performed in all 25 HPV-ind CCs and an addition 28 HPV-asso CCs. It was shown that gene expression profiles differed between HPV-asso and HPV-ind CCs. For example, HDAC9, FGFR were highly expressed in HPV-ind CCs (Fig. 3A), which was also confirmed by the TCGA database (Fig. S3A). Gene Set Enrichment Analysis (GSEA) was then performed to display the different patterns of activated pathways between these two types (Figs. 3B; S3B). The PI3K/AKT signaling pathway was significantly enriched in HPV-ind CCs in both cohorts (Fig. 3C), while the P53 signaling pathway was highly activated in HPV-asso CCs (Fig. 3D; Fig. S3C), suggesting that the PI3K/AKT signaling, instead of TP53 signaling, may strongly engage in the oncogenic effects of HPV-ind CCs. Concurrently, the fibroblast growth factors (FGFR) family, including FGFR1 and FGFR4, was highly enriched in HPV-ind CCs (Fig. 3E; Fig. S3D). Other enrichment pathways between these two CCs included the abnormal activation of G-protein coupled receptor (GPCR) and the calcium signaling pathway in HPV-ind CCs, as well as hyperactivation of the senescence-associated secretory phenotype (SASP), G2M checkpoint and E2F related signaling in HPV-asso CCs. To undercover the downstream effects of activated PI3K/AKT signaling, a gene network of FGFR-PI3K-AKT signaling pathway was constructed (Fig. 3F). In addition to the receptors FGFR1, FGFR4 and ERBB4, the main effector AKT1 and its downstream effectors, MTOR, GSK3B, p21, WEE1, BAD and CREB5, which mediate cancer cell growth and progression, also exhibited marked changes in HPV-ind CCs. Collectively, the aberrant activation of the PI3K/AKT pathway may act as a potential driver of cervical carcinogenesis in the absence of HPV infection, thus indicating the potential role of PI3K inhibitors. Furthermore, the activation of the FGFR pathway suggested a combined therapeutic role of PI3K and TKI inhibitors in HPV-ind CCs.

### Immune infiltration was suppressed in HPV-ind CCs

The tumor microenvironment (TME) was analyzed between HPV-ind and HPV-asso CCs in two cohorts. Sixty-four cell types in these two groups were quantified by the xCell algorithm, and the differences are shown in Fig. S4A and Fig. S4B. HPV-ind CCs contain more matrix cells, such as the pericytes, fibroblasts and preadipocytes. Additionally, compared to HPV-asso CCs, common immune cells such as lymphoid progenitor (CLP), pro B-cells and CD8+ naïve T-cells were less infiltrated in HPV-ind individuals. Next, immune-related pathways were further analyzed by ssGSEA (Fig. S4C, D). As expected, antigen processing- and presentation- related immune-promoted pathway were significantly downregulated, while members of the immune-inhibited pathway (TGF-β family members and their receptors) were upregulated in HPV-ind CCs. Furthermore, BCR, TCR signaling pathway, TNF family members receptors, chemokines and interferons were downregulated in HPV-ind CCs from TCGA cohort. (Fig. S4D). We next merged the two cohorts in calculating the immune cell infiltration by the CIBERSORT algorithm to acquire a higher confidence. Apparently, the HPV-ind CCs had less immune cell infiltration than the HPV-asso ones either in terms of innate immune cells or adaptive immune cells, including plasma cells, CD8 + T cells, activated CD4+ memory T cells, activated NK cells, dendritic cells, and activated macrophages, accompanied by enrichment of resting immune cells, anti-immune M2 microphage, mast cells and neutrophils. (Fig. S4E–G). Hence, a relatively suppressed immune state was exhibited in HPV-ind CCs, which also explained the worse prognosis, and indicated that HPV-ind CC patients may hardly benefit from immunotherapies such as immune checkpoint inhibitors or cancer vaccines due to the barren TME.

### PI3K-α inhibitor BYL719 has a superior effect on HPV-ind cervical cancer cells

**Fig. 4:**
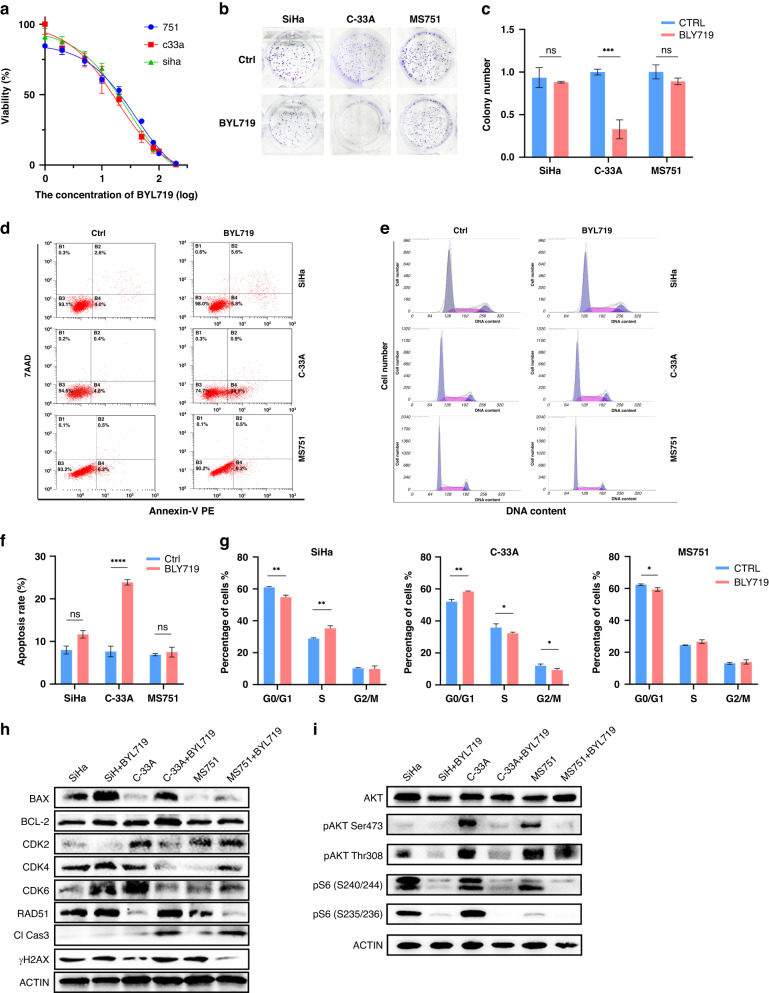
PI3K-α inhibitor BYL719 has a superior effect on HPV-ind CC cells. **A** CCK-8 assay was analyzed with BYL719 treatment for 72 h. Cells were treated with 25uM BYL719 for 10 days and then stained with crystal violet (**B**). Colonies with over 50 cells were quantified (**C**). **D** Cells were treated with BYL719 for 72 h and then stained with Annexin-V and 7-AAD. Apoptosis proportion was analyzed by flow cytometry. **E** Cells were treated with BYL719 for 72 h and then stained with PI. Cell cycle distribution was analyzed by flow cytometry. Proportion of apoptosis cells (**F**) and cell cycle (**G**) was presented as histogram. (**H**–**I**) The expression level of apoptosis, cell cycle, damage repair marker (**H**) and downstream substrates of PI3K-Akt signaling pathway (**I**) after treatment of BYL719 for 72 h determined by western blotting. All data are presented as means ± SD of three independent experiments. *P < 0.05; **P < 0.01. Wilcox test.

Considering the significant role of aberrant activation of the PI3K/AKT signaling pathway, the inhibitory effects of PI3K α-selective inhibitor BYL719 were evaluated in HPV-ind (C-33A) and HPV-asso cervical cancer cell lines (SiHa and MS751). It revealed that C-33A cells were more sensitive to BYL719 than MS751 and SiHa cells (IC50: 19.94 nmol/L (C-33A) vs. 26.69 nmol/L (SiHa); 38.39nmol/L (MS751), Fig. 4A). A concentration of 25nmol/L BYL719 significantly inhibited C-33A cell proliferation but barely affected that of SiHa and MS751 (Fig. 4B, C). Additionally, BYL719 treatment contributed to higher apoptosis rate (23.7% (C-33A) vs. 11.67% (SiHa); 7.5% (MS751), *p* < 0.001, Fig. 4D, F) as well as more cells arrest in the G0/G1 phase in C-33A cells than that of SiHa and MS751 cells (Fig. 4E, G). Consistently, corresponding changes of apoptosis, proliferation and cell cycle-related proteins were observed in C-33A cells (Fig. 4H). Increased phosphorylation of downstream substrates of the PI3K/AKT signaling pathway was significantly repressed on the treatment of BYL719 in C-33A cells (Fig. 4I), supporting our hypothesis of the aberrant activation of the PI3K/AKT signaling pathway and the inhibitory effect of BYL719 in HPV-ind CCs.

### BYL719 showed marked anti-tumor effect on HPV-ind cervical cancer patient derived models

**Fig. 5:**
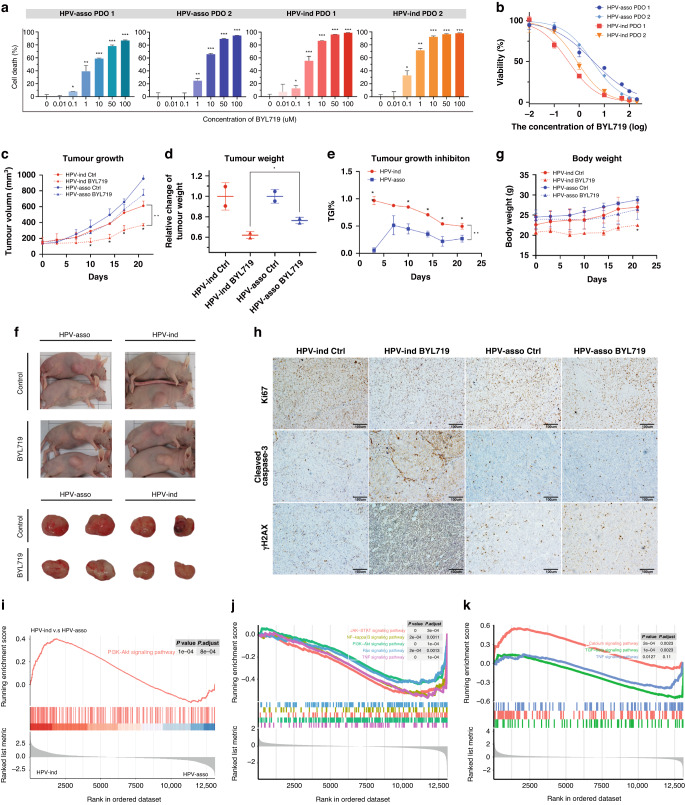
BYL719 showed marked anti-tumor effect on HPV-ind CC patient derived models. **A** Quantification of cell death in four HPV-ind and HPV-asso PDOs after 120 h of treatment with different concentrations of BYL719. **B** CCK-8 assay of HPV-ind and HPV-asso PDOs were analyzed with BYL719 treatment for 120 h. Tumor growth (**C**), tumor weight (**D**) and TGI% (**E**) curve of HPV-ind and HPV-asso PDX models treated with BYL719 p.o. for 28 days. **F** Comparison of the gross appearances of HPV-ind PDX and HPV-asso PDX between control and BYL719 treated groups (*n* = 2 samples for each group). **G** The body weight curve showed a decrease in HPV-ind PDX after 7 days of treatment with BYL719. **H** Representative images of Ki67, cleaved caspase3 and γH2AX staining of HPV-ind and HPV-asso PDX tissues. **I** GSEA for untreated PDXs showing enrichment of the PI3K/AKT signaling pathway in HPV-ind PDX. GSEA for PDXs treated with BYL719 p.o. for 28 days showing different patterns of changed tumor-related signaling pathways. Top 5 significant enriched pathways were shown for HPV-ind PDX (**J**). All 3 significant enriched pathways were shown for HPV-asso PDX (**K**). All data are presented as means ± SD of three independent experiments. *P < 0.05; **P < 0.01. Wilcox test.

Next, we evaluated BYL719 in two HPV-ind and two HPV-asso patient-derived organoids (PDOs) (Fig. 5A). Consistent with the in vitro experiments, HPV-ind PDOs had lower IC50 as compared to HPV-asso PDOs (0.59 ± 0.22 nmol/L vs. 3.54 ± 0.16 nmol/L, *P* = 0.013, Fig. 5B). Similarly, the antitumor effects of BYL719 were determined in two patient-derived tumor xenograft (PDX) models from HPV-ind and HPV-asso patients. The xenografts were treated with BYL719 for 28 days. It showed that tumor proliferation was significantly repressed with a higher tumor growth inhibition rate (TGI%) in HPV-ind PDXs than that with HPV-asso xenografts (Fig. 5C–F), however, an obvious body weight loss was also observed (Fig. 5G). Furthermore, tumors of HPV-ind PDXs treated with BYL719 displayed reduced staining of Ki67, and increased staining of cleaved caspase3 and γH2AX, indicating that BYL719 had superior effect on inhibiting tumor proliferation, promoting tumor apoptosis and DNA damage in HPV-ind PDXs than that with HPV-asso PDXs (Fig. 5H).

To further understand the effect of BYL719 on TME in HPV-ind and HPV-asso PDXs, RNA-Seq was performed with tumor tissues. It identified that the PI3K-Akt signaling pathway was highly enriched in HPV-ind PDXs compared to HPV-asso PDXs in untreated groups, although PIK3CA mutation was not carried (Fig. 5I). While it was evidently inhibited under the treatment of BYL719, the treatment also influenced the other cancer-related pathways, such as JAK-STAT, NF-kB, Ras, TNF signaling pathways (Fig. 5J). However, only changes in calcium, TNF and TGF-beta signaling pathways were found after the exposure of BYL719 in HPV-asso PDXs (Fig. 5K). In summary, these results revealed that the PI3K-Akt signaling pathway was aberrant activated in HPV-ind CCs, and these patients may benefit from the PI3K α-selective inhibitor BYL719.

### Molecular subgroups of HPV-ind CCs were identified by RNA-Seq

**Fig. 6:**
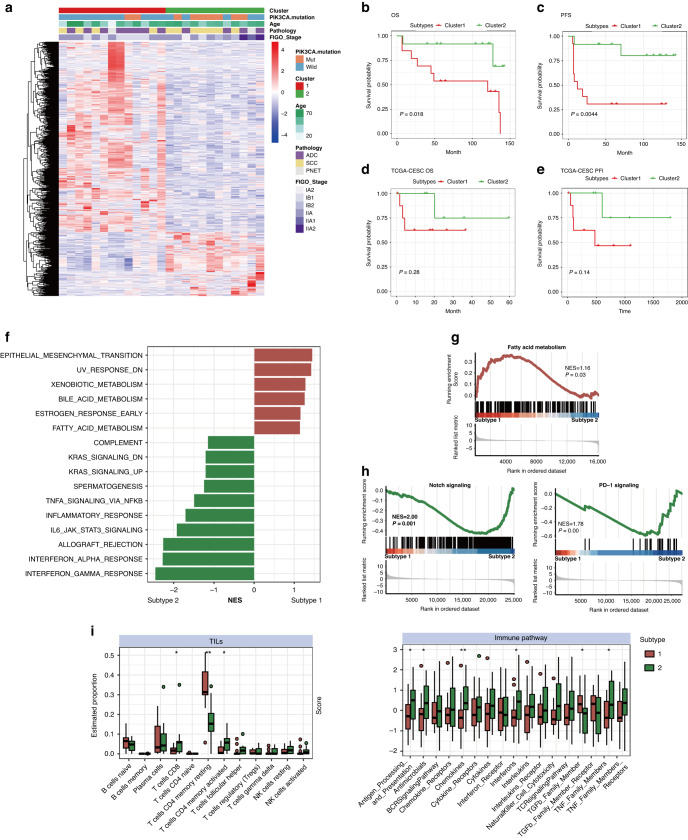
Molecular clustering of HPV-ind CCs. **A** Heatmap with clinical features annotated above of 2 clusters from the SHCC cohort identified by unsupervised hierarchical clustering. Kaplan-Meier plot in two clusters of OS (**B**) and PFS (**C**) in the SHCC cohort, OS (**D**) and PFI (**E**) in the TCGA-CESC cohort. **F** Bar plot shows cancer-related pathways enriched by GSEA between two subtypes in merged cohort. The representative GSEA plot shows that the fatty acid metabolism was enriched in subtype 1 (**G**) while the PD-1 signaling and signaling by NOTCH were enriched in subtype 2 (**H**). **I** Bar plot displays different enrichments in TILs and immune-related pathways between two subtypes in merged cohorts. NS no significance. *P < 0.05; **P < 0.01. Wilcox test.

To investigate the heterogeneity among HPV-ind CCs, our RNA-Seq data was analyzed using unsupervised hierarchical clustering to classify 25 HPV-ind CCs into different subgroups. The optimal number (*K* = 2) of clusters was chosen by ConsensusClusterPlus package. (Fig. 6A and Fig. S5A). Survival analyses of the two clusters illustrated an obviously lower survival probability for subtype 1 in terms of both OS (HR: 5.9, CI: 1.3–27.2, *P* = 0.018, Fig. 6B) and PFS (HR: 8.4, CI:1.8-39.1, *P* = 0.0044, Fig. 6C). Subsequently, the same features were also validated in the TCGA cohort (Fig. S5B, C). Survival analyses showed a difference in survival probability between the two clusters, albeit with no statistical significance in the log-rank test (Fig. 6D, E). Principal component analysis (PCA) conducted managed to differentiate the clusters in both cohorts (Fig. S5D, E), and the clinical features of the two clusters in SHCC and TCGA cohorts were depicted in Table S5

Further independent analysis of both cohorts showed that subtype 1, named metabolic subtype, was more active in metabolism and epithelial-mesenchymal transition, while immune-related pathways were distinctly enriched in subtype 2, named immune subtype (Fig. S5G, H). The results were consistent with those of separate analyses when the samples from two cohorts were combined (Figs. 6F and S5I). In detail, the metabolic subtype was enriched in fatty acid metabolism while the immune subtype was enriched with targets of novel antitumor targeted therapies, such as PD-1 signaling and Notch signaling (Fig. 6G, H), In addition, PIK3CA was found highly mutated in immune subtype in the SHCC cohort (15% (subtype1) vs. 58% (subtype2), *P* = 0.041, Fisher exact test), but there were no differences in PI3K-AKT signaling activation between these two subtypes (Fig. S5F), suggesting an alternative PI3K-AKT signaling activating mechanism beyond the PIK3CA mutation in the metabolic subtype. Furthermore, the immune subtype had more tumor-infiltrating lymphocytes (TILs) especially CD8+ and activated CD4 + T cells, and higher immune score in most immune-promoting pathways, including antigen processing and presentation, chemokines, interferons, and TNF-related pathways, alongside lower scores for the immune-suppressive TGF-β pathway. (Fig. 6I).

Collectively, we identified two subtypes with distinct biological characteristics and outcomes in HPV-ind CCs, which indicated the heterogeneity of HPV-ind CCs and personalized therapies should be suggested in different subtypes.

## Discussion

Our study revealed that HPV-ind CCs accounted for 2.48% (25/1010) of CCs with FIGO stage IB-IIA under our most stringent cutoff. It had poorer survival than that with HPV-asso CCs. The PI3K/AKT pathway was highly activated by PIK3CA, PTEN and AKT mutation in HPV-ind CCs, which may elucidate the pathogenesis of HPV-ind CCs and indicate the clinical applications of PI3Kα inhibitor in HPV-ind CCs patients. Also, HPV-ind CCs were divided into metabolic subtype and immune subtype according to different prognosis, suggesting personalized therapies in HPV-ind CCs.

Previous studies of the percentage of HPV-ind CCs fluctuated between 1%–15% [3, 6, 7, 23, 24]. The methods used for HPV detection varied, comprising hybrid capture 2 (HC2), PCR targeting HPV DNA L1 region or E6/E7 region, and commercially available kits using reverse hybridization after PCR. The detectable HPV subtypes also different from high-risk HPV to a total of 34 of high- to low- risk HPV types. However, no more than two methods were applied to HPV detection in most reports, leading to the false negatives and disparities in the prevalence of HPV-ind CCs. In this study, a new sequential procedure for HPV screening was used for two available kinds of samples (freshly frozen tissues and paraffin-embedded sections); DNA and cDNA were used for PCR, which primers were designed to target type-specific E6/E7 regions and the conserved L1 open reading frame. More importantly, RNA-Seq, capable of identifying 195 subtypes of HPV, including novel strains such as RTRX7, L55, etc. was used for validation, reducing the incidence of false positives. Consequently, the HPV-ind CCs samples screened in our study shared high accuracy, which provides necessary guarantee for the subsequent analysis of molecular characteristics of HPV-ind CCs.

In this study, it demonstrated that the prevalence of HPV-ind CCs among different histological subtypes were 1.5% (11/733) for SCC, 7.8% (12/154) for ADC, and 1.8% (2/110) in other types, including ASC, NEC and carcinosarcoma (CS), which was slightly different from what has been reported. Pirog and Holl showed that nearly all SCCs were HPV-asso, while it accounted 14% and 10% in ADC and ASC, respectively [25, 26]. Of all HPV-ind CCs in our study, 44% (11/25) were SCC, 48% (12/25) were classic ADC, and 8% (2/25) were neuroendocrine carcinoma (NEC). Thus, HPV-ind CCs were more common seen in ADC, which also explained poor survival of ADC, and encouraged us to explore the molecular type of ADC. In addition, HPV-ind CCs were associated with older onset age and poorer prognosis in both our cohort and the TCGA cohort, although the difference was not significant in the TCGA cohort due to the limited sample size. These findings led to the hypothesis that HPV-ind CCs formed a distinct type of CCs from traditional HPV-asso CCs and therefore called for an in-depth study of the pathogenesis and characteristics as well as novel potential treatment modalities.

The current study revealed integrated genomic and transcriptomic molecular features of HPV-ind CCs using WES and RNA-Seq technologies. It showed a highly mutation of genes related to pathogenic PI3K/AKT pathway in HPV-ind CCs. Notably, 52% of HPV-ind CCs had oncogene PIK3CA mutations, compared to only 14% in HPV-asso CCs. However, in HPV-ind head and neck squamous cell carcinomas (HNSCs) and anal squamous cell carcinomas, the mutation rate of PIK3CA was significantly lower than that in HPV-asso ones [27–30], while in penile carcinoma, the rate was similar [31]. Further transcriptomic analysis validated the activation of PI3K/AKT pathway caused by ERBB4 and FGFR1/4 overexpression and PTEN deletion in HPV-ind CCs. This suggests that the influenced cell proliferation, survival and glycolysis caused by aberrant activation of PI3K pathway may be the oncogenic driver event of HPV-ind CCs, which deserves more research for validation.

Given the highly activation of PI3K pathway in HPV-ind CCs, the efficacy of PI3Ka inhibitor BYL719 was evaluated in vitro and in vivo. Superior effect on HPV-ind C-33A cells than HPV-asso SiHa and MS751 cells was observed. Also, BYL719 demonstrated better antitumor effects in the HPV-ind PDO and PDX models, the latter showed an aberrant upregulation of PI3K pathway but not carrying PIK3CA mutation, than in the HPV-asso PDX. These results indicated a favorable response to PI3Kα inhibitors in HPV-ind CCs. Alpelisib (BYL719) was first approved by the FDA in 2019 for combined treatment with fulvestrant for PIK3CA mutated, HR + , HER- advanced or metastatic breast cancer [32]. It demonstrated a good efficacy in PIK3CA-altered solid tumors, especially in cervical cancer, from the first-in-human study of Alpelisib [33]. Consequently, non-HPV infectious status may be a clinical indication for BYL719 administration, which requires clinical trials for validation.

Interestingly, we also identified enrichment of FGFR pathway in HPV-ind CCs, a receptor initiates a cascade of intracellular events involved in angiogenesis, cell proliferation and cellular survival [34], as well as the downstream activation of PI3K. To today, numerous FGFR inhibitors have been developed for the malignancies with aberrant alteration of FGFR [34], among which, Erdafitibinib, Pemigatinib and Infigratinib have been approved by the FDA. Therefore, we hypothesized that HPV-ind CCs maybe a possible new indication for trials of FGFR inhibitors, although further research is required. In addition, TMB predicts immunotherapy response and was found to be significantly elevated in HPV-ind CCs, suggesting a potential role for combination immunotherapy with PI3K inhibitors.

CNV aberrations in critical regions are generally thought to be deleterious [35]. In the present study, despite that we failed to find novel CNVs in HPV-ind CCs, an imbalance in CNV levels between HPV-ind CCs and HPV-asso CCs was revealed. This may be explained by the fact that HPV virus can integrate into the host’s DNA [36]. In addition, an amplified region, 3q26.31 (8/15, 53.3%), was found in HPV-ind CCs, which were not harbored with PIK3CA mutation. Especially, FNDC3B was located and enriched in that area. Previous studies illustrated that FNDC3B abundance was correlated with the development and invasion in CCs [37], HNSCs [38] and gastric cancers [39], and it also predicted a poor prognosis [40]. Therefore, we hypothesized that FDNC3B may act as a surrogate pathogenic factor in PIK3CA wild-type HPV-ind CCs. However, more studies are needed before a conclusion can be drawn.

The present study also first presented the heterogeneity within HPV-ind CCs and divided patients into two subtypes: the immune subtype and the metabolic subtype. As termed, the immune subtype was characterized by enrichment of immune-related pathway and TILs, accompanied by a better prognosis. Our classification was also verified in the TCGA-CESC cohort. Similar to previous studies, enhanced PD-1 signaling and NOTCH signaling were found in the immune subgroup [18, 41], and both could be targeted for immunotherapy [42]. In particular, the PD-1 inhibitor, Pembrolizumab has shown great advantages in prolonging PFS and OS in persistent, recurrent, or metastatic cervical cancer patients [43]. Considering the enhanced PI3K/AKT pathway in HPV-ind CCs, a combination of a PI3K inhibitor and a PD-1/PD-L1 inhibitor may achieve a better result in the immune group [44] (NCT03711058). Whereas the metabolic subtype may benefit from metabolic inhibitors targeting fatty acid synthesis; however, more research into potential therapeutic targets is still needed.

In summary, our work has clarified the proportion of HPV-ind CCs and explored the genomic and transcriptomic characterization of this unique type. The findings suggest a highly activation of PI3K/AKT pathway, especially caused by PIK3CA mutation, in HPV-ind CCs, and emphasize the application of PI3K inhibitor for the treatment. With the increasing incidence of HPV-ind cervical cancer, further studies with larger sample size are required.

### Supplementary information

Supplementary Tables

Supplementary Materials

## Data Availability

Any additional information required to reanalyze the data reported in this paper is available from the lead contact upon request.
