# Supplementary material for: Integrated genomic and transcriptomic analysis reveals the activation of PI3K signaling pathway in HPV-independent cervical cancers
Source: Br J Cancer. 2024 Jan 22;130(6):987–1000. doi: 10.1038/s41416-023-02555-w (PMC10951256; doi:10.1038/s41416-023-02555-w)
Supplement: Supplementary file 2 — Supplementary Materials [file 41416_2023_2555_MOESM2_ESM.docx]

**Supplementary Materials and Methods**

***The detection of HPV infection***

For type-specific real-time PCR, the LCR/E6/E7 regions of the HPV genome were chosen for amplification targeting the clinically most relevant high-risk HPV types (HPV 16,18, 31, 33, 45, 52, 58) ^1^. HPV type-specific primers and corresponding TaqMan probes were designed and chosen following Lindh. M *et al^2,3^*, as presented in Table S1. Real-time PCRs were performed for 45 cycles (15 s at 94 ℃, 20 s at 50 ℃ and 40 s at 60 ℃ each) using an ABI 7900HT Real-Time PCR system (Applied Biosystems). Samples with threshold cycle (Ct) values of <35 were considered HPV positive.

For consensus primer PCRs, GP5+/6+ and PGMY9/11, targeting the conserved L1 open reading frame of the HPV genome, were used for amplification of general HPV DNA. The primer sequences were designed according to studies of Resnick *et al* and Husman *et al*^4,5^, and are presented in Table S1. The following PCR program was used: 40 amplification cycles were performed under the procedure of 95 °C for 2 min, 40 cycles of 95 °C for 1 min, 55 °C for 1 min, and 72 °C for 1 min, followed by a final extension of 10 min at 72 ℃, and then stored at 4℃. Similarly, samples with a Ct value of <35 were considered HPV positive.

The samples identified by the above methods were subjected to RNA-Seq for validation, which was described in detail below.

***Paraffin sectioning, DNA extraction and cDNA preparation***

DNA and cDNA from fresh frozen tissues has been prepared as our previous study described^6^. Formalin-fixed, paraffin-embedded tissue specimens were obtained and sectioned according to the previously described 'sandwich' procedure^7^. A series of sections were cut; the outer and inner sections were stained with H&E for histological analysis, whereas the central sections were used for DNA and RNA extraction. Part of tissues from PDX xenografts were stored in RNAlater stabilization solution for RNA isolation.

RNA and genomic DNA from paraffin-embedded slice were extracted using a DNA/RNA Isolation Kit (Tiangen Biotech), according to the standard protocol provided by the manufacturer; 2 μg of total RNA was reverse-transcribed into single-stranded cDNA using an M-MLV Reverse Transcriptase Kit (Invitrogen).

***Whole-exome sequencing (WES)***

DNA of tumor tissues and paired normal tissues from 21 HPV-ind CCs patients was subjected to WES. Genomic DNA was extracted and then sheared using the Biorupter (Diagenode, Belgium) to acquire 200–300-bp fragments. The ends of DNA fragments were repaired, and the Illumina adaptor was added. After the sequencing library was constructed, whole exomes were sequenced on an Illumina platform (Illumina, San Diego, CA) with 150-bp paired-end reads.

***Genomic alterations***

Raw reads were filtered to remove low quality reads using FastQC. Clean reads were mapped to the reference genome GRCh37 using BWA. After removing duplications, the single-nucleotide variant (SNV) and insertion and deletion (Indel) were called and annotated using the Genome Analysis Toolkit (GATK) based on dbSNP build 150^8^. All variants were annotated with ANNOVAR^9^. A series of filtering criteria were applied to the variant candidates to finally identify SNVs and Indels: (1) variants with a quality <30, a depth of coverage <20 in tumor or plasma samples, or <2 reads supporting the variant were filtered out; (2) only variants within the exonic or splicing site were retained; (3) variants reported in more than 1% of the population in the 1000 Genomes Project (1000gAUG_2015ALL) or the Exome Aggregation Consortium (ExAC_ALL) or the Genome Aggregation Database (gnomAD_ALL) in the East Asian population were discarded as they were regarded as singlenucleotide polymorphisms; and (4) synonymous variants were excluded.

For somatic mutations, we used similar in silico methods in previous studies to identify cancer-related variants, including how to determine the harmfulness of variants and annotated SNVs^10^. Several functional prediction algorithms were used to evaluate the effect of each variant, including SIFT, Polyphen2 and MutationTaster with default settings. Only variants that were predicted to be deleterious, damaging, or disease by at least one tool were retained. Indels annotated in the Catalogue of Somatic Mutations in Cancer (COSMIC v70) database were included^11^. For germline mutations, using the InterVar database, all variants were classified following the guidelines of the American College of Medical Genetics and Genomics (ACMG), which are commonly used for the interpretation of germline variants. Similar to a previous study^12^, only germline mutations classified as pathogenic, likely pathogenic, or variants of uncertain significance (VUS) based on the ACMG guidelines were retained in our study.

Somatic mutations and somatic copy number alterations for the TCGA cohort were downloaded from the GDC database. Significant somatic mutations were analyzed by MutSigCV as previous described^13^ using WES data annotated by Mutect2. Driver events of copy number alterations were obtained by using GISTIC 2.0^14^ on GenePattern (<https://cloud.genepattern.org/>). The R package maftools was used for summarization and visualization^15^. We counted the total number of nonsynonymous mutations to assess the tumor mutation burden.

***Quantification of RNA expression levels***

RNA-Seq reads were mapped onto the reference human genome GRCh38 using two-pass mapping of STAR (v2.5)^16,17^. The number of reads per gene was counted with featureCounts using the GENCODE v29 annotation^18^. Each gene expression level normalized to transcripts per-million was computed using a custom PERL script.

***Characterization of Immune infiltration of the microenvironment***

The enrichment levels of 64 immune signatures were analyzed by the xCell algorithm^19^. The relative fractions of 22 infiltrating immune cell types were quantified by the CIBERSORT algorithm^20^. The enrichment of immune-related pathways obtained from the ImmPort database(<https://immport.niaid.nih.gov>) was assessed by single-sample gene set enrichment analysis (ssGSEA).

References:

1. Myers G, D.H., Icenogle J, et al. (eds). (1994, 1995 and 1996.). Human Papillomavirus Compendium: A Compilation and Analysis of Nucleic Acid and Amino Acid Sequences. Los Alamos, NM: Los Alamos National Laboratory.

2. Lindh, M., Gorander, S., Andersson, E., Horal, P., Mattsby-Balzer, I., and Ryd, W. (2007). Real-time Taqman PCR targeting 14 human papilloma virus types. J Clin Virol 40, 321-324. 10.1016/j.jcv.2007.09.009.

3. Schmitz, M., Scheungraber, C., Herrmann, J., Teller, K., Gajda, M., Runnebaum, I.B., and Dürst, M. (2009). Quantitative multiplex PCR assay for the detection of the seven clinically most relevant high-risk HPV types. J Clin Virol 44, 302-307. 10.1016/j.jcv.2009.01.006.

4. de Roda Husman, A.M., Walboomers, J.M., van den Brule, A.J., Meijer, C.J., and Snijders, P.J. (1995). The use of general primers GP5 and GP6 elongated at their 3' ends with adjacent highly conserved sequences improves human papillomavirus detection by PCR. J Gen Virol 76 ( Pt 4), 1057-1062. 10.1099/0022-1317-76-4-1057.

5. Resnick, R.M., Cornelissen, M.T., Wright, D.K., Eichinger, G.H., Fox, H.S., ter Schegget, J., and Manos, M.M. (1990). Detection and typing of human papillomavirus in archival cervical cancer specimens by DNA amplification with consensus primers. J Natl Cancer Inst 82, 1477-1484. 10.1093/jnci/82.18.1477.

6. Xiang, L., Jiang, W., Ye, S., He, T., Pei, X., Li, J., Chan, D.W., Ngan, H.Y.S., Li, F., Tao, P., et al. (2018). ERBB2 mutation: A promising target in non-squamous cervical cancer. Gynecol Oncol 148, 311-316. 10.1016/j.ygyno.2017.12.023.

7. JAN M. M. WALBOOMERS, M.V.J. (1999). Human papillomavirus is a necessary cause of invasive cervical cancer worldwide. J Pathol 189, 12-19.

8. DePristo, M.A., Banks, E., Poplin, R., Garimella, K.V., Maguire, J.R., Hartl, C., Philippakis, A.A., del Angel, G., Rivas, M.A., Hanna, M., et al. (2011). A framework for variation discovery and genotyping using next-generation DNA sequencing data. Nat Genet 43, 491-498. 10.1038/ng.806.

9. Wang, K., Li, M., and Hakonarson, H. (2010). ANNOVAR: functional annotation of genetic variants from high-throughput sequencing data. Nucleic Acids Res 38, e164. 10.1093/nar/gkq603.

10. Sun, S., Thorson, J.A., and Murray, S.S. (2019). Annotation of Variant Data from High-Throughput DNA Sequencing from Tumor Specimens: Filtering Strategies to Identify Driver Mutations. Methods Mol Biol 1908, 49-60. 10.1007/978-1-4939-9004-7_4.

11. Forbes, S.A., Beare, D., Boutselakis, H., Bamford, S., Bindal, N., Tate, J., Cole, C.G., Ward, S., Dawson, E., Ponting, L., et al. (2017). COSMIC: somatic cancer genetics at high-resolution. Nucleic Acids Res 45, D777-d783. 10.1093/nar/gkw1121.

12. Lebeault, M., Pinson, S., Guillaud-Bataille, M., Gimenez-Roqueplo, A.P., Carrie, A., Barbu, V., Pigny, P., Bezieau, S., Rey, J.M., Delvincourt, C., et al. (2017). Nationwide French Study of RET Variants Detected from 2003 to 2013 Suggests a Possible Influence of Polymorphisms as Modifiers. Thyroid 27, 1511-1522. 10.1089/thy.2016.0399.

13. Lawrence, M.S., Stojanov, P., Polak, P., Kryukov, G.V., Cibulskis, K., Sivachenko, A., Carter, S.L., Stewart, C., Mermel, C.H., Roberts, S.A., et al. (2013). Mutational heterogeneity in cancer and the search for new cancer-associated genes. Nature 499, 214-218. 10.1038/nature12213.

14. Mermel, C.H., Schumacher, S.E., Hill, B., Meyerson, M.L., Beroukhim, R., and Getz, G. (2011). GISTIC2.0 facilitates sensitive and confident localization of the targets of focal somatic copy-number alteration in human cancers. Genome Biol 12, R41. 10.1186/gb-2011-12-4-r41.

15. Mayakonda, A., Lin, D.C., Assenov, Y., Plass, C., and Koeffler, H.P. (2018). Maftools: efficient and comprehensive analysis of somatic variants in cancer. Genome Res 28, 1747-1756. 10.1101/gr.239244.118.

16. Zheng, Q., Zhao, J., Yu, H., Zong, H., He, X., Zhao, Y., Li, Y., Wang, Y., Bao, Y., Li, Y., et al. (2020). Tumor-Specific Transcripts Are Frequently Expressed in Hepatocellular Carcinoma With Clinical Implication and Potential Function. Hepatology 71, 259-274. 10.1002/hep.30805.

17. Dobin, A., Davis, C.A., Schlesinger, F., Drenkow, J., Zaleski, C., Jha, S., Batut, P., Chaisson, M., and Gingeras, T.R. (2013). STAR: ultrafast universal RNA-seq aligner. Bioinformatics 29, 15-21. 10.1093/bioinformatics/bts635.

18. Liao, Y., Smyth, G.K., and Shi, W. (2014). featureCounts: an efficient general purpose program for assigning sequence reads to genomic features. Bioinformatics 30, 923-930. 10.1093/bioinformatics/btt656.

19. Aran, D., Hu, Z., and Butte, A.J. (2017). xCell: digitally portraying the tissue cellular heterogeneity landscape. Genome Biol 18, 220. 10.1186/s13059-017-1349-1.

20. Newman, A.M., Liu, C.L., Green, M.R., Gentles, A.J., Feng, W., Xu, Y., Hoang, C.D., Diehn, M., and Alizadeh, A.A. (2015). Robust enumeration of cell subsets from tissue expression profiles. Nat Methods 12, 453-457. 10.1038/nmeth.3337.

**
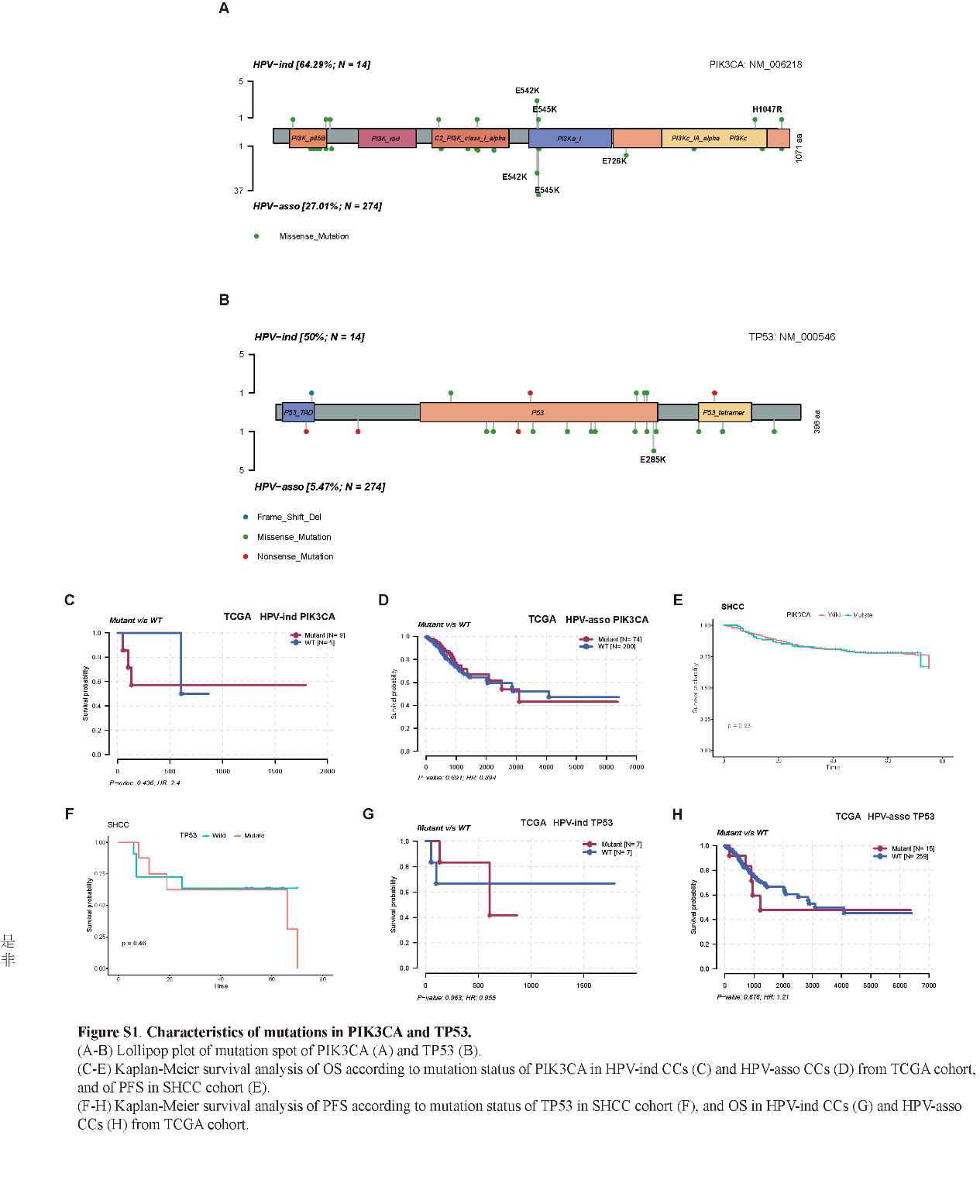
**

**Fig. S1 Characteristics of mutations in PIK3CA and TP53.**

**A-B** Lollipop plot of mutation spot of PIK3CA (A) and TP53 (B). **C-E** Kaplan-Meier survival analysis of OS according to mutation status of PIK3CA in HPV-ind CCs (C) and HPV-asso CCs (D) from TCGA cohort, and of PFS in SHCC cohort (E). **F-H** Kaplan-Meier survival analysis of PFS according to mutation status of TP53 in SHCC cohort (F), and OS in HPV-ind CCs (G) and HPV-asso CCs (H) from TCGA cohort.


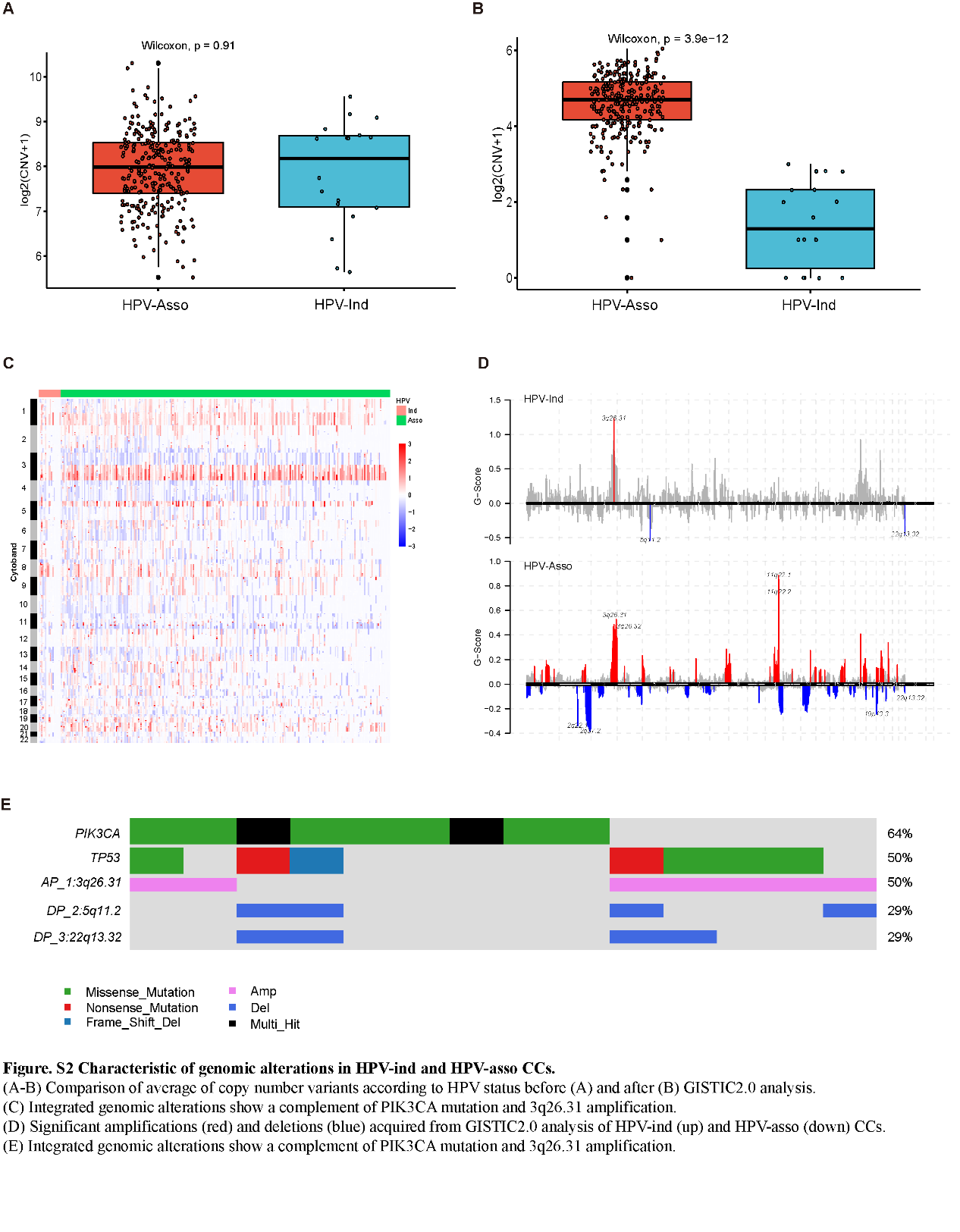


**Fig. S2 CNV landscape of HPV-asso and HPV-ind CCs.**

**A-B** Comparison of the average of copy number variants according to HPV status before (A) and after (B) GISTIC2.0 analysis. **C** Global CNV profile of the TCGA-CESC cohort. **D** Significant amplifications (red) and deletions (blue) acquired from GISTIC2.0 analysis of HPV-ind (up) and HPV-asso (down) CCs. **E** Integrated genomic alterations show a complement of PIK3CA mutation and 3q26.31 amplification.


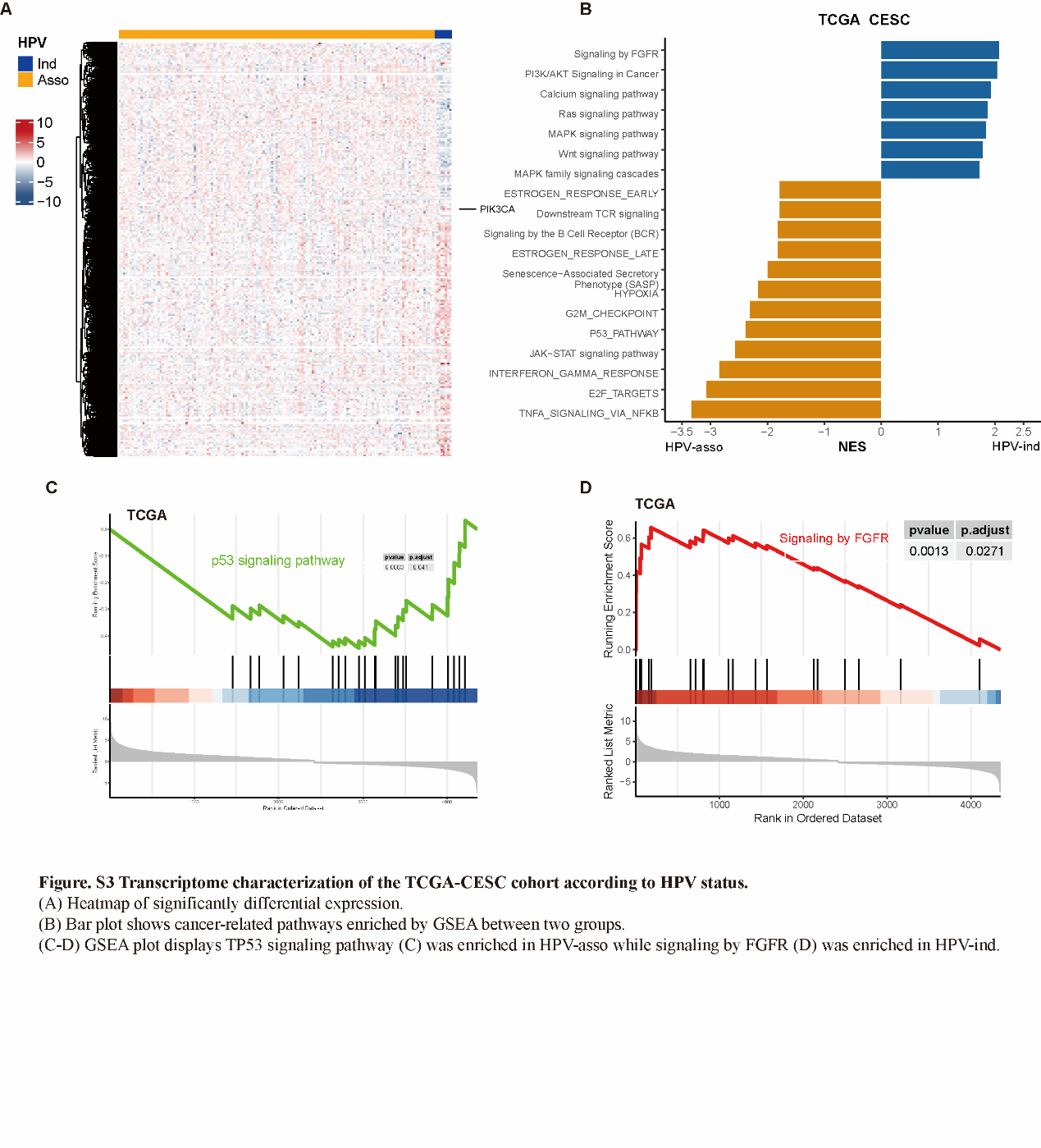


**Fig. S3 Transcriptome characterization of the TCGA-CESC cohort according to HPV status.**

**A** Heatmap of significantly differential expression. **B** Bar plot shows cancer-related pathways enriched by GSEA between two groups. **C-D** GSEA plot displays TP53 signaling pathway (C) was enriched in HPV-asso CCs while signaling by FGFR (D) was enriched in HPV-ind CCs.


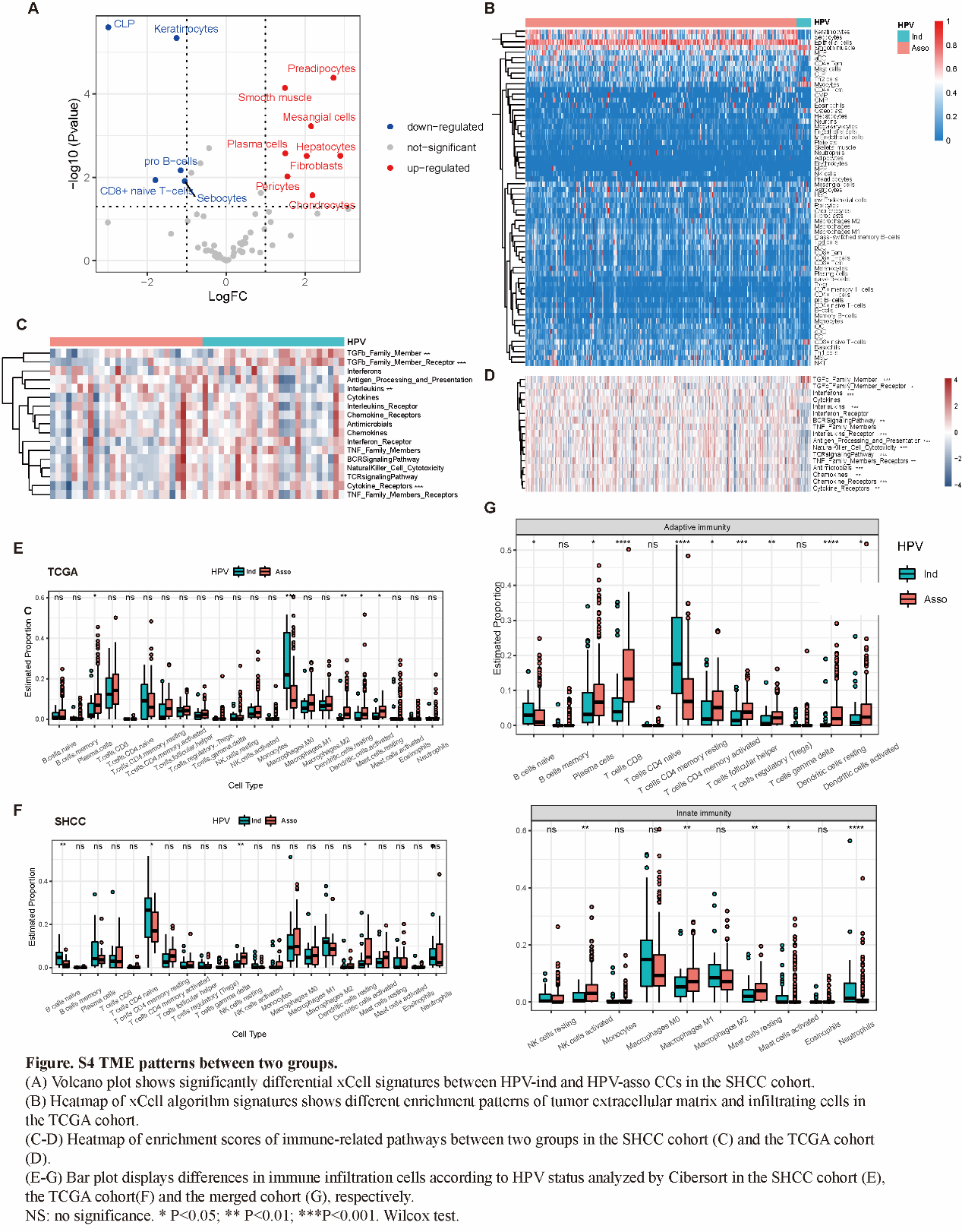


**Fig. S4 TME patterns between two groups.**

**A** Volcano plot shows significantly differential xCell signatures between HPV-ind and HPV-asso CCs in the SHCC cohort**. B** Heatmap of xCell algorithm signatures shows different enrichment patterns of tumor extracellular matrix and infiltrating cells in the TCGA cohort. **C-D** Heatmap of enrichment scores of immune-related pathways between two groups in the SHCC cohort (C) and the TCGA cohort (D). **E-G** Bar plot displays differences in immune infiltration cells according to HPV status analyzed by Cibersort in the SHCC cohort (E), the TCGA cohort(F) and the merged cohort (G), respectively. NS: no significance. * P<0.05; ** P<0.01; ***P<0.001. Wilcox test.


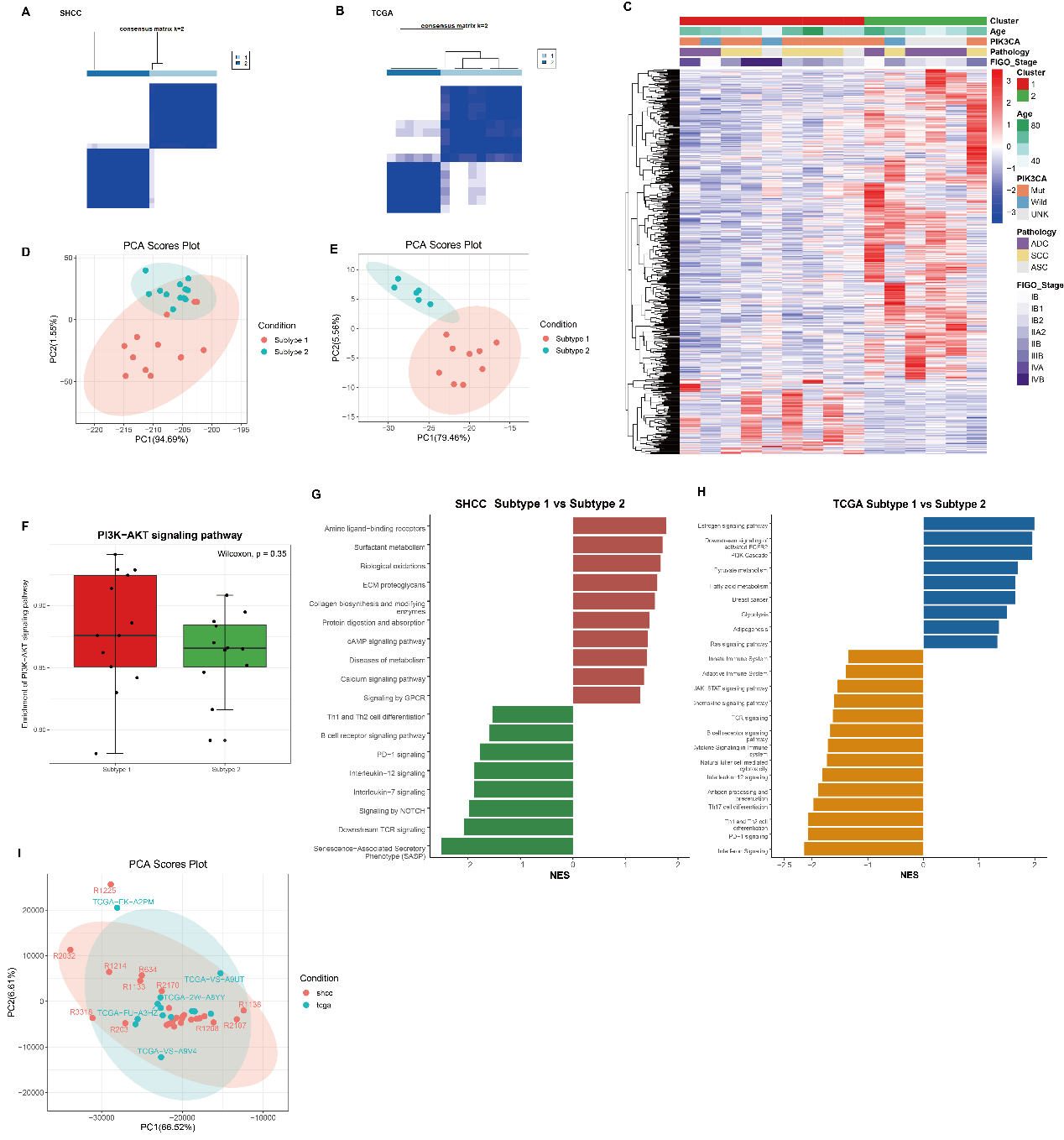
**Fig.S5 Molecular clustering of HPV-ind CCs using RNA-Seq.**

**A-B** Consensus matrix of unsupervised hierarchical clustering when K=2 in SHCC (A) and TCGA-CESC (B) cohorts. **C** Heatmap with clinical features annotated above of 2 clusters from the TCGA cohort identified by unsupervised hierarchical clustering. **D-E** Two-dimensional scatterplot of Principal Component Analysis (PCA) within the two clusters in SHCC (D) and TCGA-CESC (E) cohorts. **F** Boxplot of the enrichment score of the PI3K-AKT signaling pathway showing no differences of the PI3K/AKT signaling pathway between 2 subtypes from the SHCC cohort. The enrichment score was acquired by ssGSEA. **G-H** Bar plot shows cancer-related pathways enriched by GSEA between two subtypes in the SHCC cohort (G) and the TCGA cohort (H). **I** Two-dimensional scatterplot of PCA within HPV- CCs shows no batch effect between the TCGA and the SHCC cohorts
